# Supplementary material for: Low effect of young afforestations on bird communities inhabiting heterogeneous Mediterranean cropland
Source: PeerJ. 2015 Dec 7;3:e1453. doi: 10.7717/peerj.1453 (PMC4675097; doi:10.7717/peerj.1453)
Supplement: Supplemental Information 1 [file peerj-03-1453-s001.docx]

**Supplementary Material**

**Table S1**. List of all species surveyed at 80 1000 m long x 200 m wide transects located on farmland habitat adjacent to tree plantations in Central Spain. For each species, the following information is provided for the entire transect and for two segments of the transect (i.e. < 400 m or close to tree plantations and > 600 m or away for plantations): frequency (number of transects where the species occurred), local abundance (number of individuals km^-2^, mean ± sd), regional abundance at six farmland habitat types in the Mesomediterranean region in the breeding season (number of individuals km^-2^, mean ± sd; source: Carrascal and Palomino 2008), SPEC (source: BirdLife International 2004b), inclusion in the European farmland bird index (EFBI, + if included, source: Directorate-General for Agriculture and Rural Development 2012), and inclusion in the list of common farmland birds in Southern Europe (CFBSE, + if included, source: European Bird Census Council). The study area was not part of the distribution area of 10 species included in the EFBI list (*Ciconia ciconia, Corvus frugilegus, Emberiza citrinella, Emberiza melanocephala, Lanius collurio, Lanius minor, Limosa limosa, Perdix perdix, Saxicola rubetra* and *Sturnus vulgaris*) and six species included in the CFMSE list (the former but *C. frugilegus, E. citrinella, L. limosa* and *S. rubetra*). * indicates the species that were not considered for statistical analyses in this study.

| Species | Winter | | | | Breeding season | | | | Regional abundance | SPEC | EFBI | CFBSE |
| --- | --- | --- | --- | --- | --- | --- | --- | --- | --- | --- | --- | --- |
|  | Close | | Away | | Close | | Away | |  |  |  |  |
|  | Freq. | Abund. | Freq. | Abund. | Freq. | Abund. | Freq. | Abund. |  |  |  |  |
| *Accipiter gentilis** |  |  | 1 | 5.0 |  |  |  |  |  | Non |  |  |
| *Actitis hypoleucos** | 1 | 5.0 |  |  |  |  |  |  |  | 3 |  |  |
| *Aegithalos caudatus* |  |  | 1 | 5.0 |  |  |  |  |  | Non |  |  |
| *Alauda arvensis* | 12 | 25.8±28.8 | 12 | 35.8±51.1 | 6 | 6.7±2.6 | 5 | 9.0±6.5 | 2.5±1.7 | 3 | + | + |
| *Alectoris rufa* | 11 | 8.6±3.9 | 11 | 8.2±4.0 | 13 | 8.5±3.8 | 10 | 8.0±3.5 | 34.5±21.6 | 2 |  | + |
| *Anthus pratensis* | 4 | 153.8±168.5 | 6 | 60.0±90.3 |  |  |  |  |  | Non | + |  |
| *Apus apus** |  |  |  |  | 6 | 15.0±11.4 | 5 | 20.0±12.7 |  | Non |  |  |
| *Aquila adalberti** |  |  | 1 | 5.0 |  |  |  |  |  | 1 |  |  |
| *Athene noctua** |  |  |  |  |  |  | 2 | 5.0±0.0 |  | 3 |  |  |
| *Bubulcus ibis* |  |  | 1 | 5.0 |  |  |  |  |  | Non |  |  |
| *Burhinus oedicnemus* |  |  | 1 | 5.0 | 3 | 5.0±0.0 | 6 | 6.7±2.6 | 2.0±1.8 | 3 | + | + |
| *Buteo buteo** |  |  | 3 | 5.0±0.0 |  |  |  |  |  | Non |  |  |
| *Carduelis cannabina* | 17 | 74.4±116.0 | 25 | 62.6±125.6 | 32 | 8.3±4.7 | 27 | 12.6±11.2 | 46.2±21.6 | 2 | + | + |
| *Carduelis carduelis* | 31 | 21.6±26.5 | 37 | 71.2±199.7 | 19 | 11.3±8.5 | 22 | 8.6±4.1 | 77.8±46.4 | Non |  | + |
| *Carduelis chloris* | 2 | 5.0±0.0 | 2 | 10.0±0.0 |  |  | 1 | 10.0 | 44.8±37.1 | Non |  | + |
| *Carduelis spinus* |  |  | 1 | 5.0 |  |  |  |  |  | Non |  |  |
| *Circus aeruginosus** | 1 | 5.0 | 1 | 5.0 | 1 | 5 | 2 | 5.0±0.0 |  | Non |  | + |
| *Clamator glandarius* |  |  |  |  |  |  | 1 | 5.0 | 1.3±0.8 | Non |  |  |
| *Columba livia* | 4 | 50.0±38.5 | 10 | 56.5±110.7 | 14 | 41.4±45.8 | 13 | 21.0±20.0 | 26.5±14.0 | Non |  | + |
| *Columba oenas* | 1 | 75.0 | 3 | 11.7±7.6 | 0 |  | 1 | 5.0 | 0.4±0.6 | Non |  |  |
| *Columba palumbus* | 13 | 30.0±59.4 | 9 | 11.1±3.3 | 29 | 8.4±4.0 | 27 | 9.8±6.1 | 30.2±11.4 | Non |  |  |
| *Corvus corone* |  |  | 1 | 60.0 |  |  |  |  | 0.8±0.3 | Non | + | + |
| *Coturnix coturnix* | 1 | 5.0 |  |  |  |  | 1 | 5.0 | 0.9±0.5 | 3 |  |  |
| *Cyanistes caeruleus* |  |  | 2 | 7.5±3.5 |  |  |  |  |  | Non |  |  |
| *Delichon urbicum** |  |  |  |  | 1 | 5.0 |  |  | 21.0±24.2 | 3 |  | + |
| *Emberiza calandra* |  |  | 1 | 5.0 | 9 | 8.3±5.0 | 4 | 6.3±2.5 | 57.4±33.3 | 2 | + | + |
| *Emberiza cirlus* |  |  | 2 | 82.5±95.5 |  |  |  |  | 2.4±4.4 | 1 | + | + |
| *Emberiza hortulana* |  |  |  |  |  |  | 1 | 10.0 | 0.2±0.3 | 2 | + | + |
| *Emberiza schoeniclus* | 1 | 15.0 |  |  |  |  |  |  |  | Non |  |  |
| *Erithacus rubecula* | 2 | 5.0±0.0 | 1 | 5.0 |  |  |  |  |  | Non |  |  |
| *Falco naumanni** |  |  |  |  | 1 | 5.0 | 1 | 5.0 |  | 1 |  |  |
| *Falco tinnunculus** | 3 | 5.0±0.0 | 1 | 5.0 | 2 | 10.0±7.1 | 2 | 10.0±0.0 | 5.8±2.7 | 3 | + | + |
| *Fringilla coelebs* | 16 | 25.3±37.2 | 21 | 66.2±114.6 | 3 | 6.7±2.9 | 5 | 10.0±6.1 | 37.5±40.2 | Non |  |  |
| *Galerida cristata* | 23 | 10.0±6.9 | 19 | 11.8±8.2 | 36 | 10.1±6.9 | 34 | 7.9±4.6 | 135.8±57.6 | 3 | + | + |
| *Hirunda daurica** |  |  |  |  | 2 | 10.0±0.0 |  |  |  | Non |  |  |
| *Hirundo rustica** |  |  |  |  | 6 | 15.0±9.5 | 5 | 6.0±2.2 | 83.0±40.4 | 3 | + | + |
| *Lanius meridionalis* | 3 | 6.7±2.9 | 4 | 5.0±0.0 | 2 | 12.5±10.6 | 2 | 5.0±0.0 | 3.7±1.9 | 3 |  |  |
| *Lanius senator* |  |  |  |  | 1 | 5.0 | 3 | 5.0±0.0 | 10.4±7.3 | 2 | + | + |
| *Lophophanes cristatus* |  |  |  |  |  |  | 1 | 5.0 |  | 2 |  |  |
| *Melanocorypha calandra* | 14 | 32.9±66.0 | 10 | 31.0±48.8 | 10 | 8.0±3.5 | 7 | 7.9±5.7 | 28.6±39.4 | 3 | + | + |
| *Merops apiaster** |  |  |  |  |  |  | 5 | 10.0±5.0 | 19.2±9.8 | 3 |  | + |
| *Milvus migrans** |  |  |  |  | 1 | 10.0 | 1 | 5.0 |  | 3 |  |  |
| *Milvus milvus** | 1 | 10.0 | 3 | 6.7±2.9 |  |  |  |  |  | 2 |  |  |
| *Motacilla alba* | 5 | 10.0±6.1 | 6 | 70.0±101.1 | 1 | 5.0 |  |  | 6.5±5.2 | Non |  | + |
| *Oenanthe hispanica* |  |  |  |  | 1 | 5.0 | 1 | 5.0 | 2.8±2.5 | 2 | + | + |
| *Parus major* | 6 | 11.7±6.1 | 2 | 5.0±0.0 | 2 | 5.0±0.0 | 2 | 5.0±0.0 | 24.6±22.3 | Non |  |  |
| *Passer domesticus* | 1 | 180.0 | 3 | 80.0±39.1 | 4 | 11.3±9.5 | 3 | 20.0±13.2 | 337:9±114.1 | 3 |  | + |
| *Passer hispaniolensis* |  |  | 1 | 10.0 | 1 | 10.0 |  |  | 9.5±12.2 | Non |  |  |
| *Passer montanus* |  |  |  |  | 1 | 5.0 |  |  | 12.6±19.7 | 3 | + | + |
| *Pernis apivorus** |  |  |  |  | 1 | 120.0 | 1 | 5.0 |  | Non |  |  |
| *Petronia petronia* | 1 | 5.0 | 2 | 7.5±3.5 |  |  |  |  | 5.9±4.8 | Non | + | + |
| *Phoenicurus ochruros* | 3 | 8.3±5.8 | 5 | 5.0±0.0 |  |  |  |  |  | Non |  |  |
| *Phylloscopus collybita/ P. ibericus* | 6 | 6.7±4.1 | 10 | 6.0±3.2 |  |  |  |  |  | Non |  |  |
| *Pica pica* | 13 | 11.5±8.5 | 29 | 11.9±9.3 | 22 | 9.1±6.1 | 24 | 9.4±7.0 | 31.6±17.8 | Non |  | + |
| *Pluvialis apricaria* | 1 | 65.0 |  |  |  |  |  |  |  | Non |  |  |
| *Pterocles alchata* | 1 | 40.0 | 1 | 55.0 | 8 | 16.9±11.3 | 10 | 19.0±23.8 |  | 3 |  |  |
| *Pterocles orientalis* |  |  | 7 | 10.7±4.5 | 3 | 10.0±0.0 | 1 | 20.0 |  | 3 |  |  |
| *Saxicola torquata* | 5 | 7.0±2.7 | 2 | 5.0±0.0 | 1 | 5.0 |  |  | 11.6±6.2 | Non | + | + |
| *Serinus serinus* | 5 | 46.0±51.2 | 14 | 67.1±77.1 | 3 | 11.7±2.9 | 3 | 15.0±0.0 | 95.6±105.8 | Non | + | + |
| *Streptopelia decaocto* | 2 | 7.5±3.5 |  |  | 5 | 7.0±2.7 | 3 | 6.7±2.9 | 7.4±2.7 | Non |  |  |
| *Streptopelia turtur* |  |  |  |  | 3 | 8.3±2.9 | 2 | 5.0±0.0 | 7.6±6.6 | 3 | + | + |
| *Sturnus unicolor* | 6 | 46.7±87.7 | 10 | 44.5±31.7 | 8 | 10.0±6.5 | 10 | 43.5±44.7 | 179.7±83.3 | Non | + | + |
| *Sylvia atricapilla* | 4 | 5.0±0.0 | 2 | 7.5±3.5 |  |  |  |  |  | Non |  |  |
| *Sylvia melonocephala* |  |  | 1 | 5.0 |  |  |  |  |  | Non |  |  |
| *Sylvia undata* | 1 | 5.0 |  |  |  |  |  |  |  | 2 |  |  |
| *Tetrax tetrax* | 1 | 15.0 | 4 | 357.5±480.0 | 13 | 7.3±3.9 | 8 | 28.1±40.8 |  | 1 |  |  |
| *Turdus merula* | 2 | 5.0±0.0 | 2 | 10.0±0.0 |  |  | 1 | 5.0 | 27.5±26.0 | Non |  |  |
| *Turdus philomelos* | 2 | 5.0±0.0 | 3 | 11.7±2.9 |  |  |  |  |  | Non |  |  |
| *Turdus viscivorus* |  |  | 1 | 5.0 |  |  |  |  |  | Non |  |  |
| *Upupa epops* | 1 | 20.0±0.0 |  |  | 2 | 5.0±0.0 | 2 | 5.0±0.0 | 5.2±2.4 | 3 | + | + |
| *Vanellus vanellus* |  |  |  |  | 1 | 5.0 |  |  |  | 2 | + | + |
